# Biologia Futura: is ADAM 17 the reason for COVID-19 susceptibility in hyperglycemic and diabetic patients?

**DOI:** 10.1007/s42977-021-00092-2

**Published:** 2021-06-08

**Authors:** Ganna Stepanova

**Affiliations:** Faculty of Medicine, Institute of Translational Medicine, Semmelweis University, Budapest, Hungary

**Keywords:** COVID-19, SARS-CoV-2, Diabetes, Hyperglycemia, RAS inhibitors, ADAM17, Insulin, Heme oxygenase (HO)-1

## Abstract

COVID-19 is a disease-causing current pandemic. It prevails in patients with pre-existing conditions such as diabetes and hypertension. Renin–angiotensin system was identified as a center of COVID-19 pathophysiology. There is a current controversy concerning the usage of ACE inhibitors and AR blockers in patients with COVID-19. Multiple clinical trials are on the way to determine the effect of RAS blockers in patients with COVID-19. ACE2 receptor is thought to be the point of entry utilized by a coronavirus. However, other factors have been identified which potentially facilitate SARS-CoV-2 entry into the cell. ADAM17 could facilitate viral entry in hyperglycemic and diabetic patients. Insulin is an ADAM17 inhibitor. Heme oxygenase (HO)-1 level is reduced in diabetic patients, contributing to the worst outcome for patients with poor glycemic control. The combined therapy of glycemic control and antioxidant response to oxidative stress could be explored in patients with COVID-19.

## Novelty and innovation

According to recent findings, there is still a controversy present regarding the usage of ACEi and ARBs in patients who tested positive for new coronavirus infection (Kessler and Schunkert 2020). The controversy is that ACEi and ARB can increase the expression of ACE2 receptors on the surface of cells (Roncati et al. 2020). Scientists worldwide are also trying to determine why some populations are prone to the new coronavirus infection to implement appropriate treatment. This review addresses alternative possibilities for COVID-19 pathophysiology and treatment options for patients with and without diabetes mellitus.

## Background

### Coronavirus disease 2019

Coronavirus disease 2019 (COVID-19) is caused by Severe Acute Respiratory Syndrome Coronavirus 2 (SARS-CoV-2), which was identified in December 2019 in Wuhan, China, and spread all over the world, leading to a pandemic. (https://apps.who.int/iris/handle/10665/330376)

COVID-19 symptoms and diseases related to the effect of the action of SARS-CoV-2 on RAS listed in Cao et al. article are impacting lungs, heart, kidney, liver, testis, central nervous (CNS), digestive system, eye, skin, blood vessels, adipose tissue, promotes thrombosis/coagulopathy (effect on blood coagulation), dysfunction of immunity/differentiation and activation of immune cells (e.g., monocytes-macrophages), autoimmune diseases (e.g., multiple sclerosis, chronic inflammatory demyelinating polyneuropathy, Miller Fisher syndrome), and even cancer (effect on cell proliferation and migration, as well as the production of pro-inflammatory mediators, including adhesion molecules) (Cao et al. 2020).

### A brief overview of the renin–angiotensin system (RAS)

Renin–angiotensin system (RAS) signaling, specifically angiotensin-converting enzyme 2 (ACE2), was identified in the pathogenesis of COVID-19. ACE 2 receptor is associated with infection with SARS-CoV-2 and was initially proposed as the main point of viral entry into the cell (Zhang et al. 2020).

**Fig. 1:**
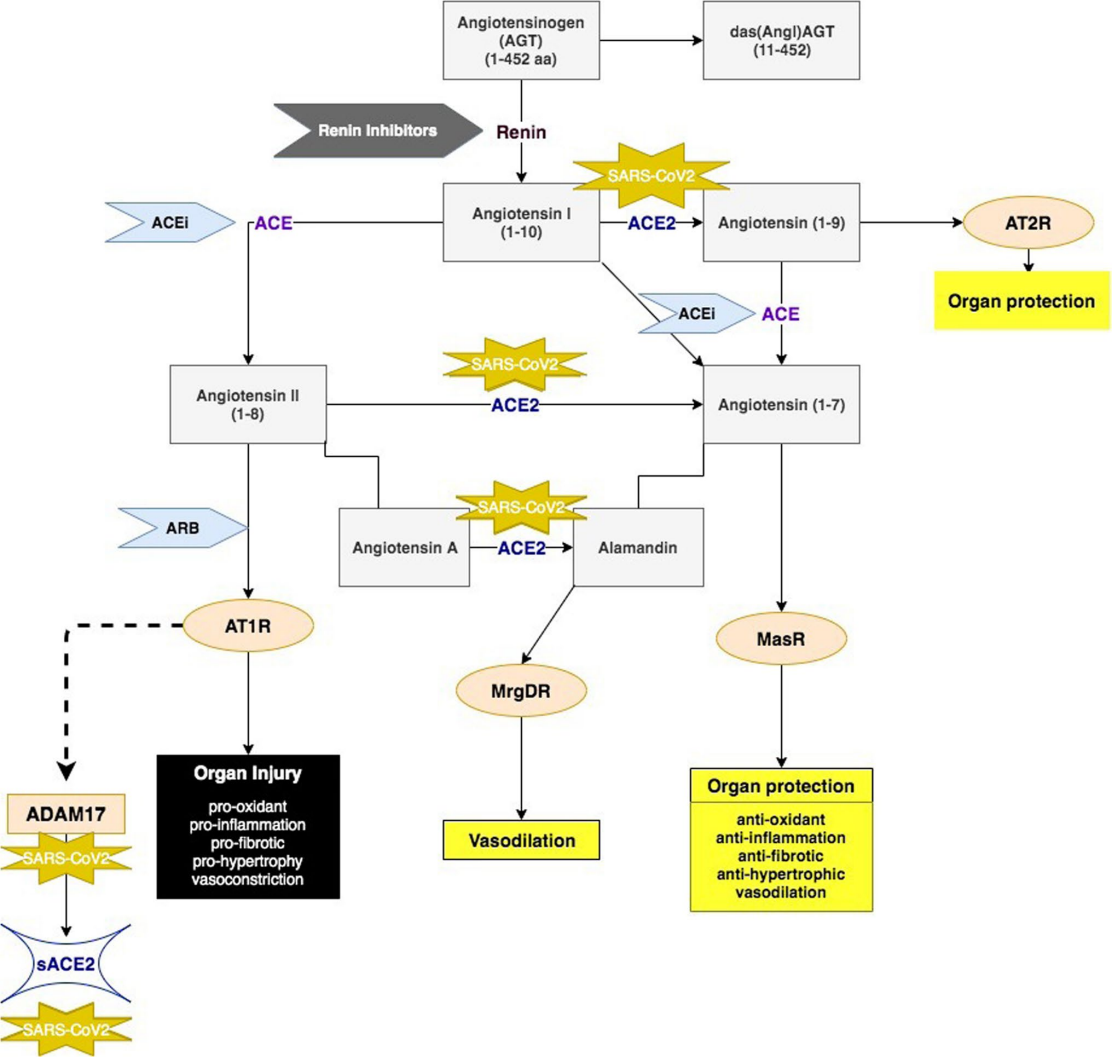
*Renin–angiotensin system (RAS).* Angiotensinogen (AGT) (1–452 amino acids) disassociates to das (Ang I) AGT and Ang I. Ang I further spits to active metabolites acting on receptors promoting organ injury or protection. SARS-CoV2 binds to the ACE2 receptor and ADAM17. AT1R activates ADAM17, which subsequently promotes ACE2 shedding. ACE inhibitors suppress Ang I’s conversion to A II and Ang (1–9) to Ang (1–7). AR blockers stop the binding of Ang II to AT1R

RAS (RAAS -including aldosterone) is a complex cascade of angiotensinogen (AGT) dissociation to more minor constituents (Fig. 1). First disassociation forms two metabolites Angiotensin I (1–10) and des (Ang I) AGT (11–485). Angiotensin I (Ang I) is the source of active angiotensin peptides in various physiological functions. The biological property of des (Ang I) AGT is mostly unknown (Lu et al. 2016).

ACE2 is involved in converting Ang I to Angiotensin (1–7), which activates Mas 1 oncogene receptor (MasR). ACE2 also converts Ang I to Angiotensin (1–9), which eventually becomes Ang (1–7). Additionally, ACE2 converts Angiotensin A (Ala-Ang(1–8)) to Alamandine (Ala-Ang(1–7)), leading to the activation of MAS-related G protein-coupled receptor D receptor (MrgD), which positively regulates vasodilation (Schleifenbaum 2019).

### ACEi/ARB

Angiotensin-converting enzyme inhibitors (ACEi) are suppressing the conversion of Ang I–Ang II, Ang (1–9) to Ang (1–7), Ang (1–7)-Ang (1–5). Angiotensin receptor blockers (ARB) hinder the activation of angiotensin receptor 1 (AT1R). Angiotensin II accumulation’s detrimental effect while activating the ACE-Ang II-AT1R pathway is aggravating the progression of the disease-promoting oxidation, inflammation, organ hypertrophy, vasoconstriction, and fibrosis (Gurwitz 2020).

The potential difference between ACEi and ARB administration outcomes may arise from both drugs’ mechanism of action. It was suggested that suppression of Ang (1–7) formation by ACEi might reduce the positive effect of MasR pathway activation. MasR pathway plays an antioxidant, anti-inflammatory, anti-fibrotic, anti-hypertrophic role, and reduces reactive oxygen species (ROS), and promoting vasodilation (Meng et al. 2020; (Omar 2020). However, it was noted by Abajo et al. that in terms of the primary outcome in COVID-19, no difference was found between ACEi and ARB administering patients, which was most notable when comparing monotherapy with these drugs (de Abajo et al. 2020).

Both ACEi and ARB are the primary chosen medications for managing hypertension and diabetes in adults (Quinn et al. 2020).

### The potential role of ADAM17

There is a notion that the AT1R positively regulates ADAM17 (CD156b; cSVP; MGC71942; tumor necrosis factor converting enzyme (TACE)), a type I transmembrane protein belonging to the adamalysin subfamily of Zn-dependent metalloproteases (Danser, Epstein, and Batlle 2020). ADAM17 has ubiquitous applications in human disease. It is involved in inflammation, sepsis, psoriasis, CNS diseases, malignancies, diabetes, atherosclerosis, angiogenesis, kidney and heart diseases (Gooz 2010). The most well-known function of catalytically active ADAM17 is to cleave ectodomains of various transmembrane proteins, such as heparin-binding epidermal growth factor (HB-EGF), which, in turn, activates EGFR and initiates cell proliferation.

This metalloprotease promotes the shedding of the ACE2 receptor from the membrane to the cytosol, resulting in soluble ACE2 (sACE2), potentially having a protective effect from the virus (Danser et al. 2020; Gheblawi et al. 2020). Phase II clinical trials were completed in January 2021 for the recombinant human angiotensin-converting enzyme 2 (rhACE2), APN01, as an intravenous treatment for patients with COVID-19 to block viral entry and decrease viral replication (ClinicalTrials.gov, clinical trial ID: NCT04335136).

Theoretically, blocking the AT1 receptor with ABR upregulation of ADAM17 might be interrupted. It was shown that a doubling of soluble ACE2 persists in cerebrospinal fluid of hypertensive patients due to the upregulation of ADAM17 in the brain (Xu et al. 2017). Nevertheless, most COVID-19 patients present hypertension, and we do not see the protective effect of this phenomenon. Since most ACE2 is located on the membrane surface, even a doubling is unlikely to significantly relocate ACE2 from the membrane to the extracellular space.

On the contrary, ACE2 shedding promotes RAS overactivation and drives cardiovascular pathogenesis. Loss of ACE2 results in Angiotensin II accumulation, leading to activation of AT1R and a cascade of p38 mitogen-activated protein kinase (MAPK)/ADAM17 phosphorylation by NAPDH oxidase 2-induced ROS formation. ADAM17 phosphorylation enhances its catalytic activity resulting in a detrimental positive feedback loop (Gheblawi et al. 2020).

Another research proposes targeting ADAM17, as it potentially facilitates viral entry into the cell (Palau et al. 2020). They referenced Haga et al. (2008) findings, where siRNA knock-down of ADAM17 reduced SARS-CoV viral entry into the cell. Recent research confirmed similarity in host receptor utilization between SARS-CoV and SARS-CoV-2, so it is safe to predict that inhibition of ADAM 17 might influence the SARS-CoV-2 capabilities of infecting the cell (Jaimes et al. 2020). Haga et al. also pointed that induction of ADAM17 by SARS-CoV was coupled with tumor necrosis factor (TNF)-alpha production, an inflammatory marker. Endothelial cell injury activates various pro-inflammatory cytokines such as interleukin IL-1, IL-6, and TNF-alpha. The increase in inflammation contributes to microvascular thrombosis.

Besides the ACE2 receptor, other factors may be involved in the entry of SARS-CoV-2, such as TMPRSS2, which is cleaving and activating the spike protein (S) of SARS-CoV2, a sialic acid receptor on the extracellular matrix of the cell, heparan sulfate (HS) (Clausen et al. 2020), CD147 and DPP4 (CD26) (clinical trial ID: NCT04382794, completed in July 2020) interacting with hemoglobin molecules located on erythrocytes or erythrocyte precursors (Sardu et al. 2020), neuropilin-1 (NRP1) expressed on the endothelial cells of the colon, urinary bladder, kidney, heart muscle, placenta, and testis (Cantuti-Castelvetri et al. 2020; Daly et al. 2020), cell-surface receptor glucose-regulated protein 78 (GRP78) (Ibrahim et al. 2020). AT2R (angiotensin II receptor type 2) recently reported interaction with ACE2 and is highly expressed in the lung (Cui et al. 2020).

### Diabetes and ADAM17

According to Renzhong et al., high glucose dose-dependently increased ADAM17 promoter activity, transcript, and protein levels in human and animal models (Li et al. 2015).

Individuals with diabetes and hypertension are at increased risk for viral infections, including the COVID-19 (Drucker 2020). ACE2 expression reduction in diabetic cardiovascular and renal systems plays a role in further disbalancing RAS by SARS-CoV-2 infection (Gheblawi et al. 2020). We hypothesize that ADAM17 elevated activity could be one reason for the high occurrence of COVID-19 disease in hyperglycemic and diabetic patients.

Recent presentations of severe COVID-19 patient outcomes with and without diabetes are associated with hematological manifestations. Prolongation of prothrombin time (PT) and activated partial thromboplastin time (aPTT), the elevation of D-dimer, fibrinogen, and fibrin degradation products (FDP), and decreased levels of antithrombin III are associated with a more severe outcome in COVID-19 patients. Other biochemical markers associated with a more severe presentation are elevations of lactate dehydrogenase (LDH), C-reactive protein (CRP), procalcitonin, Troponin T, creatinine, and liver enzymes (Kasinathan and Sathar 2020). Elevated serum ferritin is associated with high mortality in patients with COVID-19. The administration of recombinant human TNF-alpha was also demonstrated to result in hypoferremia in humans, accompanied by an increase in circulating ferritin concentration and a decrease in soluble transferrin receptor level. Iron chelating agents in case of “iron overload” will be explored in clinical trials to reduce the severity of the disease manifestations in patients with mild-to-severe COVID-19 infection (clinical trial ID: NCT04333550).

Hyperglycemia, even without the presence of diabetes, was reported to be associated with the worst outcomes for COVID-19 patients. Ceriello et al. described several possibilities of how hyperglycemia may affect the disease’s progression, such as increased insulin resistance due to inflammation and pancreatic beta-cell damage (Ceriello et al. 2020).

Prolonged hyperglycemia is associated with hemolysis. Injury-derived free heme promotes adhesion molecule expression, leukocyte recruitment, vascular permeabilization, platelet activation, complement activation, thrombosis, and fibrosis. Heme can be degraded by the anti-inflammatory enzyme heme oxygenase (HO)-1, generating biliverdin/bilirubin, iron/ferritin, and carbon monoxide. (Wagener et al. 2020). HO-1 mRNA in whole blood was lower in type 2 diabetes mellitus than controls in a population of diabetic patients living in Juana Koslay City, San Luis, Argentina (Siewert et al. 2013), suggesting the accumulation of free heme, leading to pulmonary edema, ER stress, and fibrosis. Frank Wagener et al. (2020) proposed to explore strategies to elevate HO-1 levels in patients suffering from COVID-19. They speculated that newly found treatment for severely ill COVID-19 patients dexamethasone attenuated the severity of the disease by reducing hemolysis and elevation of HO-1 levels. Together with the proper sugar management, the elevation of HO-1 provides an intriguing option for COVID-19 disease treatment. Unfortunately, an open-label clinical trial in mechanically ventilated adult patients with established moderate-to-severe ARDS caused by confirmed COVID-19 infection, admitted in a network of Spanish ICUs, to verify the efficacy of dexamethasone treatment was terminated (Clinical trial ID: NCT04325061) due to lack of enrolment. In addition, warning about the usage of glucocorticoids in patients with mild symptoms (Salem 2020) and early administration of dexamethasone were associated with the aggravated disease (Callejas Rubio et al. 2021). Therefore, glucocorticoid treatment should be reserved for patients with severe disease.

Nevertheless, the notion of targeting the antioxidant system in diabetic patients is still the right idea. Intriguingly, insulin increased the HO-1 mRNA and protein expressions in mouse renal cells (Harrison et al. 2006).

Insulin is an ADAM17 inhibitor. It was shown in animal studies that insulin normalized renal expression of ACE2 and ADAM17 in Type 1 diabetic Akita mice (Salem et al. 2014). Renal ACE2/ADAM17 and urinary ACE2 were increased in Type 2 diabetic mice researched by the same group previously. It was noted that patients with Type 1 diabetes have a shallow frequency of COVID-19 infection despite being considered a fragile population (Tatti et al. 2020; Pitocco et al. 2020). Insulin is the number one treatment for Type 1 diabetes.

Insulin resistance syndrome prevails in patients with Type 2 diabetes. Type 2 diabetes occurrence also accompanies several problems such as obesity, hypertension, and high cholesterol. A large cohort study of ~7,300 patients in China concluded that patients with Type 2 diabetes had the worst outcome for COVID-19 (Zhu et al. 2020). A recent review of hyperglycemic drugs’ usage concluded that early administration of insulin might have a protective effect against COVID-19 and attenuate lung injury (Nakhleh and Shehadeh 2020) if there is a possibility to reduce insulin resistance in patients.

The importance of proper medication choice for diabetic patients with COVID-19 is crucial since some may tamper with glucose control and worsen the patients’ outcome. Drugs like corticosteroids and a combination of antivirals lopinavir/ritonavir are frequently administered in patients with COVID-19. Type 1 interferons might damage beta-cells of the pancreas; hydroxyquinone in combination with azithromycin and macrolide antibiotics might increase dysglycemia and should be used with caution in diabetic patients as well as incompatible with insulin treatment (Pal and Bhadada 2020a). However, it was also proposed that the drug acts by raising intracellular pH that inhibits enzymatic degradation of insulin, resulting in recirculation of a substantial proportion of insulin in active form. Hydroxychloroquine also reduces pro-inflammatory cytokines, notably TNF-alpha and IL-6, and decreases insulin resistance. Hydroxychloroquine has also been found to be effective against SARS-CoV-2 in-vitro (Pal and Bhadada 2020b). However, hydroxychloroquine has failed to improve the mortality rate in the United Kingdom Recovery trial (Clinical Trial ID: NCT04381936). Insulin itself can cause hypoglycemia if administered in high doses. Glucocorticoids exert their hyperglycemic effects by reducing insulin sensitivity and insulin secretion, interfering with glucagon-like peptide-1 (GLP-1) effects, and enhancing glucagon production.

### Where could we be in 5–10 years?

The debate with regard to the usage of RAS blockers in COVID-19 is ongoing. Multiple animal studies are inconsistent, and data from human studies show no evidence of elevation of ACE2 in patients administering ACEi/ARB (Sriram and Insel 2020).

To address the dilemma of the ACEi/ARB effect, another study analyzed the gene expression of ACE2, Transmembrane Serine Protease 2 (TMPRSS2—a cofactor for viral entry) ADAM17, ACE, and AGTR1 (encodes for AT1 receptor) in lung tissue sample databases (Milne et al. 2020). ACEi use was associated with significantly lower ACE2 and TMPRSS2 expression but was not associated with ADAM17 expression, nor ARBs were associated with altered expression of these genes when studied in lung tissue. ACEi use was not associated with ACE or AGTR1 gene expression, whereas ARBs were associated with increased ACE and decreased AGTR1 gene expression. No available data on protein expression limit the study results, although the authors claim a direct correlation between protein expression and gene expression.

The current agreement is not to take patients who are already administering ACEi and ARB as medications (or switching them to alternative ACEi and ARB) for existing conditions from the medications due to COVID-19 positive tests (Quinn et al. 2020; Danser et al. 2020).

More information is needed from clinical observations. One of the recent studies in the US testing association of SARS-CoV-2 infection and usage of ACEi and ARB found no correlation between drug use and test positivity for the new coronavirus (Mehta et al. 2020). A similar outcome has been reported by the Spanish colleagues, who reported no increase in hospital admissions for COVID-19 amongst RAAS inhibitor users (de Abajo et al. 2020) nor increased mortality in RAAS inhibitor users in Korea (Jung et al. 2020). Another study from China reports that ACEi/ARB treatments were superior to other antihypertensive therapies in reducing inflammatory high-sensitivity C-reactive protein (hsCRP) and procalcitonin levels in COVID-19 patients with pre-existing hypertension (Yang et al. 2020). Besides, the inpatient use of ACEi/ARB was associated with a lower mortality rate in patients with COVID-19 presenting with GI symptoms, when typically GI symptoms are related to high mortality incidents (Tan et al. 2020).

Clinical trials are on the way for investigation of RAS inhibitors in patients with COVID-19 in the USA (clinical trial ID: NCT04338009, Elimination or Prolongation of ACE Inhibitors and ARB), Denmark (clinical trial ID: NCT04351581, Effects of Discontinuation or Continuation ACEi/ARB), Italy (clinical trial ID: NCT04331574, Observational Study to Associate Hypertension and Hypertension Treatment in COVID-19), Austria (clinical trial ID: NCT04353596, completed in February 2021, Discontinuation of ACEi/ARB), Saudi Arabia (clinical trial ID: NCT04357535, completed in November 2020 (Hakeam et al. 2020), Observations Study of the impact of ACEi and/or ARBs on the Prognosis of Patients with COVID-19), Ukraine (clinical trial ID: NCT04364984, Observational Study of Hypertensive Patients with COVID-19 receiving ACEi or ARB or DRis), Spain (clinical trial ID: NCT04367883, Observational Evaluation of Influenza Vaccination and Treatment with ACEi and RAIII in the Evolution of SARS-COVID-19 Infection), France (clinical trial ID: NCT04374695, Observational Study to Analyze the Associations between COVID-19, Hypertension, and Treatments with ACEi and ARBs/ NCT04329195, The Randomized ACORES-2 study: ACE inhibitors or ARBs Discontinuation for Clinical Outcome Risk Reduction in Patients Hospitalized for the Endemic Severe Acute Respiratory Syndrome Coronavirus (SARS-CoV-2) Infection) and others (Speth 2020).

Investigation of ADAM17 upregulation in COVID-19 patients, a comparison of the outcomes of diabetic patients with and without insulin (or different brands of insulin) treatments for the management of hyperglycemia, might answer the dilemma of appropriate sugar maintenance and possibly save lives.

Other branches of the RAS blocker system for treating patients with COVID-19 and pre-existing comorbidities should be explored, such as renin (Ding et al. 2018) and Rho A/Rho kinase system inhibitors (Calò et al. 2020).
